# The NOX toolbox: validating the role of NADPH oxidases in physiology and disease

**DOI:** 10.1007/s00018-012-1010-9

**Published:** 2012-05-31

**Authors:** Sebastian Altenhöfer, Pamela W. M. Kleikers, Kim A. Radermacher, Peter Scheurer, J. J. Rob Hermans, Paul Schiffers, Heidi Ho, Kirstin Wingler, Harald H. H. W. Schmidt

**Affiliations:** 1 Department of Pharmacology, Cardiovascular Research Institute Maastricht (CARIM), Vascular Drug Discovery Group, Faculty of Medicine, Health and Life Science, Maastricht University, Universiteitssingel 50, 6229 ER Maastricht, The Netherlands; 2 Vasopharm GmbH, Würzburg, Germany; 3 National Stroke Research Institute, Melbourne, VIC Australia

**Keywords:** siRNA, Antibodies, NADPH oxidase inhibitor, NOX4, VAS2870, NOX4 knock-out

## Abstract

Reactive oxygen species (ROS) are cellular signals but also disease triggers; their relative excess (oxidative stress) or shortage (reductive stress) compared to reducing equivalents are potentially deleterious. This may explain why antioxidants fail to combat diseases that correlate with oxidative stress. Instead, targeting of disease-relevant enzymatic ROS sources that leaves physiological ROS signaling unaffected may be more beneficial. NADPH oxidases are the only known enzyme family with the sole function to produce ROS. Of the catalytic NADPH oxidase subunits (NOX), NOX4 is the most widely distributed isoform. We provide here a critical review of the currently available experimental tools to assess the role of NOX and especially NOX4, i.e. knock-out mice, siRNAs, antibodies, and pharmacological inhibitors. We then focus on the characterization of the small molecule NADPH oxidase inhibitor, VAS2870, in vitro and in vivo, its specificity, selectivity, and possible mechanism of action. Finally, we discuss the validation of NOX4 as a potential therapeutic target for indications including stroke, heart failure, and fibrosis.

## Oxidative stress: the need for validated targets and therapeutic specificity

Reactive oxygen species (ROS) have long been suspected as being ‘bad guys’. They are frequently associated with the development and progression of chronic, degenerative, cancerous and inflammatory diseases. Indeed an excess of ROS, i.e. *oxidative stress*, caused by an imbalance between ROS production and their removal by antioxidant systems, may be a common underlying pathogenic mechanism in these diseases. With the recent additional description of possible roles of ROS in diverse physiological signaling processes another form of imbalance deserves attention, i.e. *reductive stress*—the excess of reducing agents in a cell that leads to shortage of ROS. These and other phenomena [1] may explain the poor outcomes of antioxidant therapies in clinical studies where even deleterious effects of untargeted antioxidant treatment have been reported [2–10]. Rather than attempting to systemically scavenge ROS, it may be more effective to specifically target the different enzymatic sources of pathophysiologically relevant ROS. Nevertheless, until this has resulted in clinical benefits, the oxidative stress hypothesis remains unproven.

Several ROS producing enzyme systems exist, including xanthine oxidase [11], the mitochondrial respiratory chain [12], lipid peroxidases [13], cytochrome P450 enzymes [14], and uncoupled endothelial NO synthase [15]. However, these enzymes produce ROS secondary to their damage, which can be proteolysis but is often caused by oxidative stress itself [11, 15]. Thus, there would still be the need to identify this primary source of oxidative stress. The only enzyme family known to produce ROS as their primary and sole function are NADPH oxidases. These multi-protein complexes are comprised of a catalytic, transmembrane-spanning subunit (NOX), as well as several structural and regulatory proteins localized in both the membrane and the cytosol.

## The NADPH oxidase family

**Fig. 1:**
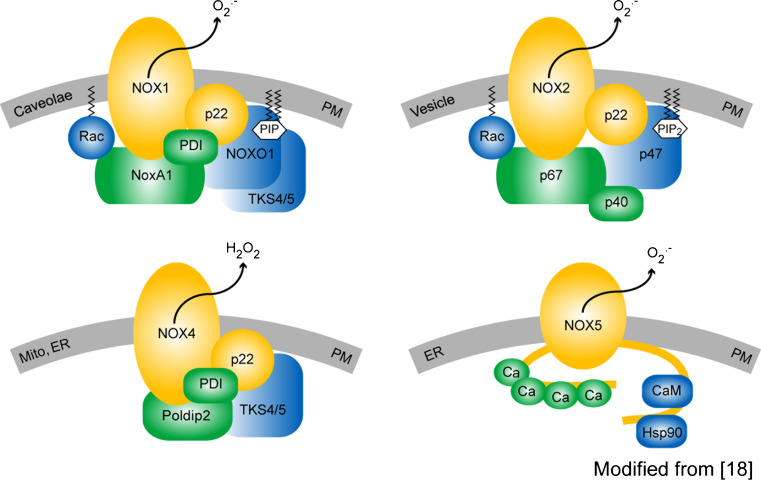
The vascular NOX isoform-based NADPH oxidase complexes. Cell or subcellular compartment membranes are shown in *gray*, core proteins in *yellow*, activator binding proteins in *green* and organizer binding proteins in *blue*. All the NOX isoforms shown are membrane proteins and are localized in the plasma membrane (PM). Additionally, NOX1 was found at the plasma membrane in caveolae [147], NOX2 in membranes of phagosomes, and NOX4 in mitochondrial [182] and ER-membranes [191], as well as in the nucleus [97]. Little is known about subcellular localization of NOX5 other than the plasma membrane, but a localization at the ER membrane has been reported [29, 192]. NOX1, NOX2, and NOX4 are associated with p22^phox^, but only NOX1 and NOX2 are regulated by the small GTPase Rac. For its activation, the NOX1 enzyme complex requires the assembly of NOX organiser 1 (NOXO1) and NOX activator 1 (NOXA1), but also forms complexes with p47^phox^ and p67^phox^ (not shown). The NOX2 enzyme complex requires binding of p47^phox^, p67^phox^, and optionally p40^phox^ that can further support the activity. In contrast to NOX1 and NOX2, NOX4 and NOX5 do not depend on any of the ‘classical’ cytosolic NADPH oxidase subunits. Recently, the protein polymerase (DNA-directed) delta-interacting protein 2 (Poldip2) was identified to bind and to increase the activity of NOX4. Further, protein disulfide isomerase (PDI) [23] and a p47^phox^ analogue tyrosine kinase substrate with 4/5 SH3 domains (Tks4/5) have been reported to bind and activate NOX1 and NOX4 [21, 22]. NOX4 is the only isoform that produces hydrogen peroxide instead of superoxide [17]. The NOX5 protein contains four N-terminal calcium-binding sites that regulate activation of the enzyme. Activity of NOX5 can be further supported by the binding of Hsp90 or Calmodulin to the C-terminus of the protein [24]

We are only beginning to understand the enzyme family of NADPH oxidases, their players and their interaction. The NOX family consists of seven members, NOX1–5, and two dual oxidases (Duox), Duox1 and Duox2. Of those, NOX1, 2, 4, and 5 have been implicated in vascular diseases, on which we focus in this review. All NOX isoforms have six trans-membrane spanning alpha helices with cytosolic N- and C-termini. They are differentially expressed and regulated in various tissues and have different subcellular localizations, and even different ROS products, i.e. superoxide versus hydrogen peroxide (reviewed in [16]). NOX1, NOX2, and NOX5 appear to produce mainly superoxide NOX4, mainly H_2_O_2_ [17]. All NOX isoforms have been reported to bind to one or more membrane and/or cytosolic proteins. p22^phox^ appears to be a general binding partner for NOX1-4 in the membrane. NOX1 and 2 also bind the small GTPase, Rac. Moreover, NOX1 binds the cytosolic subunits, NOX organizer 1 (NOXO1) and NOX activator 1 (NOXA1), and NOX2 binds the respective homologues, p47^phox^ and p67^phox^, and also the cytosolic protein, p40^phox^ [18, 19]. NOX4 was reported to bind to the polymerase (DNA-directed) delta-interacting protein 2 (PolDip2) [20]. In addition to these established NOX binding partners, the tyrosine kinase substrate with 4/5 SH3 domains (Tks4/5) [21, 22], and protein disulfide isomerase (PDI) were recently suggested to bind to both NOX1 and 4 [23]. Upon overexpression in cells, the C-terminus of NOX5 was shown to interact with Hsp90, which may also bind to NOX1 and 2 [24]. However, the physiologic relevance of these new potential binding partners for NOX function needs to be further analyzed (Fig. 1).

With respect to activity regulation, there are fundamental differences between the individual NOX catalytic subunits. Most seem to be dynamically switched on and off by either regulatory subunits (NOXA1 for NOX1 [25–27], p67phox for NOX2 [28], and calmodulin for NOX 5 [29, 30]) or intramolecularly by the N-terminal EF hands that bind free intracellular calcium (NOX5 and Duox1/2 [31]). In contrast, NOX4 is constitutively active, and modulation of its expression may thus be a major activity regulator.

## The tools to validate the role of NADPH oxidase in health and disease

During the validation of the involvement of a protein in a biological process or disease mechanism pharmacological inhibition or genetic deletion are frequently applied. In addition, specific antibodies are required to confirm the expressional regulation of NOX in a given cell or subcellular compartment. With respect to NOX biology these tools include genetic knock-out [32–35] and transgenic animals [32, 36, 37], pharmacological inhibitors, and siRNAs (see Table 1).

**Table 1:** NOX4 siRNA approaches: this table provides a selection of published siRNAs used for downregulation of NOX4

NOX isoform	Species	Sequence	Degree of NOX4 down-regulation (% of ctr.)	Ref./source	Comment
NOX4	Bovine	5′-AAGACCTGGCCAGTATATATTAT-3′	n.q. (protein)	[94]	
NOX4	Human	5′-GAGAACAGACCUGACUAUG-3′	75–85 % (protein), ~10 % (mRNA)	[95, 96]	Tested vs. NOX1 and NOX2
NOX4	Human	5′-GUUCUUAACCUCAAGUGCATT-3′ (sense); 5′-UGCAGUUGAGGUUUAAGAACTT-3′ (antisense)	n.q. (protein, mRNA)	[97]	Base error according to database sequence
NOX4	Human	5′-UUAUUGCAUAUGUAGAGGCUGUGAU-3′ (sense); 5′AUCACAGCCUCUACAUAUGCAAUAA-3′ (antisense)	n.q. (mRNA)	[98]	
NOX4	Human	5′-GUCAACAUCCAGCUGUACCdtdt-3′ (sense), 5′-GGUACAGCUGGAUGUUGACdtdt-3′ (antisense)	20 % (mRNA)	[99]	
NOX4	Human	Target sequence 5′-CAG TGA ACT ATA GTG AAC ATT TCC T-3′	40 % (mRNA)	[100]	vs. NOX2
NOX4	Human	Pool of 4: (1) ACUAUGAUAUCUUCUGGUA; (2) GAAAUUAUCCCAAGCUGUA; (3) GGGCUAGGAUUGUGUCUAA; (4) GAUCACAGCCUCUACAUAU	n.q. (mRNA)	Dharmacon [101]	
NOX4	Human	Targets exon2	40 % (mRNA and protein)	Ambion [102, 48]	ID #118807
NOX4	Human	5′-XCACCACCACCACCACCATT-3′; 5′-AAUGGUGGUGGUGGUGGUGTT-3′	n.s.	[103]	
NOX4	Human	n.s.	n.q. (protein)	Qiagen [104]	Hs_NOX4_1 and Hs_NOX4_2 predesigned
NOX4	Human	5′-CAGAACATTCCATATTAC-3 & 5′-ACTTTGTTGAACTGAATG-3′	n.q. (mRNA)	[105]	
NOX4	Human	Mixture of: (1) 5′-AAAGCAGGACAU UCAUGGAGAGCCA-3′ (sense); 5′-UGGCUCUCCAUGAAUGUCCUGGCUUU-3′ (antisense); (2) 5′-GCAUCUGUUCUUAACCUCA-3′ (sense); 5′-UGAGGUUAAGAACAGAUGC-3′ (antisense); (3) 5′-CCAGGAGAUUGUUGGAUAA-3′ (sense); 5′-UUAUCCAACAAUCUCCUGG-3′ (antisense); (4) 5′-CAGUGAAGACUUUGUUGAACUGAAU-3′ (sense); 5′-AUUCAGUUCAACAAAGUCUUCACUG-3′ (antisense)	~40 % (mRNA)	[106]	Sequences not present in NOX1, NOX2, NOX3, and NOX5
NOX4	Human	5′-AGACCUGGCCAGUAUAUUA-3′	~30 % (mRNA)	[107]	
NOX4	Human	n.s.	~38 % (mRNA) n.q. (protein)	[108]	Tested vs. NOX1, NOX2, NOX3
NOX4	Human	NOX4, 5_-CCU CUU CUU UGU CUU CUA C dTdT-3_ corresponding to nucleotides 585–603	~33 % (mRNA) ~75 % (protein)	[109]	
NOX4	Human	5′-CGAGAUGAGGAUCCUAGAAdTdT-3′ (sense); 5′-UUCUAGGAUCCUCAUCUCGdTdT-3′ (antisense)	~25 % (mRNA)	[90]	
NOX4	Human	5′-GGUACAGCUGGAUGUUGAC-3′	50 % (mRNA) n.q. (protein)	[92]	
NOX4	Human	5′-AAACCGGCAGGAGUUUACCCAG-3′	~45 % (protein) n.q. mRNA	[110, 111]	
NOX4	Human	5′-GTCAACATCCAGCTGTACCdTdT-3′	n.q. (mRNA)	[112, 113]	
NOX4	Human	(1) 5′-GATCCGCAGAACATTCCATATTACTICAAGAGAGTAATA TGGAATGTTCTGCTTTTTTGGAAA-3′ (2) 5′-GATTCCGACTTTGTTGAACTGAATGTTCAAGAGACATTC AGTTCAACAAAGTCTTTTTTGGAAA-3′	n.q. (mRNA and protein)	[114]	
NOX4	Human	(1) 5′-GAAUUACAGUGAAGACUUU-3′ (sense); 5′-AAAGUCUUCACUGUAAUUC-3′ (antisense); (2) 5′-CAGGAGGGCUGCUGAAGUA-3′ (sense); 5′-UACUUCAGCAGCCCUCCUG-3′ (antisense); (3) 5′-GGGCUAGGAUUGUGUCUAA-3′ (sense);5′-UUAGACACAAUCCUAGCCC-3′ (antisense); (4) 5′-GAUCACAGCCUCUACAUAU-3′ (sense); 5′-AUAUGUAGAGGCUGUGAUC-3′ (antisense)	n.q. (protein)	[93]	(1) and (2) not efficient, (4) most efficient
NOX4	Human, rat	5′-ACUGAGGUACAGCUGGAUGUU-3′	50 % (mRNA) n.q. (protein)	[115, 116]	NOX5 not affected
NOX4	Mouse	5′-GAC CUG ACU UUG UGA ACA UTT-3′ (sense); 5′-AUG UUC ACA AAG UCA GGU CTT-3′ (antisense)	30 % (NOX activity, protein, mRNA)	[47, 117]	Tested vs. NOX1 recommended
NOX4	Mouse	5′-GGCCAACGAAGGGGUUAAACACCUC-3′	n.q. (mRNA)	[118, 119]	
NOX4	Mouse	5′-GGAUAAAAGCAAGACUCUACACAUC-3′	(mRNA)	[119]	
NOX4	Mouse	Mix of 3 siRNAs: 5′-CCAUUUGCAUCGAUACUAA-3′; 5′-CCAAGACUCUUCAUAGUUU-3′; 5′-CAAGACCUCUCUCCUUUGA-3′	40 % (mRNA)	Santa Cruz [120]	
NOX4	Mouse	Target sequence: 5′-CAGGAATAAATTAAAGCTTTA-3′	n.s.	[121]	
NOX4	Mouse	28-kDa NOX4 (5′-AATGTTGGGCTGTCCTACTGA-3′ (sense) UGUUGGGCUGUCCUACUGAdTdT (antisense), UCAGUAGGACAGCCCAACAdTdT and full-length 65 kDa and 28 kDa (5′-AACGAAGGGGTTAAACACCTC-3′ and 5′-AAAAGCAAGACTCTACACATC-3′)	80 % (mRNA), 60 % (protein)	[43]	
NOX4	Mouse	n.s.	18 % (mRNA)	Santa Cruz [122]	vs. NOX2
NOX4	Mouse	n.s.	n.q. (protein)	Ambion [123, 124]	ID #184259 and #184261
NOX4	Mouse	Pool of 3–5 siRNAs	n.q. (mRNA and protein)	Santa Cruz [125]	# sc-41587
NOX4	Mouse	(1) 5′-AACGAAGGGGTTAAACACCTC-3′, (2) 5′-AAAAGCAAGACTCTACACATC-3′	n.q. (protein)	[126]	
NOX4	Mouse	5′-GGUUACAGCUUCUACCUAC-3′ (sense); 5′-GUAGGUAGAAGCUGUAACC-3′ (antisense)	n.q. (protein and mRNA)	Dharmacon [93]	In vivo treatment
NOX4	Pig	n.s.	50-60% (protein)	Dharmacon [127]	Tested vs. NOX2
NOX4	Rat	siRNA against Nox4 5′-AACGAAGGGGTTAAACACCTC-3′	~40 % (mRNA), n.q. (protein)	[128]	Tested vs. NOX1
NOX4	Rat	n.s.	~50 % (protein and mRNA)	Dharmacon [129]	
NOX4	Rat	5′-GUAGGAGACUGGACAGAAA-3′ (sense)	n.d.	[130]	
NOX4	Rat	(1) 5′-GUUAGUCUGUGUGUGGCUGtt-3′, (2) GAUUUGCCUGGAAGAACCCtt-3′	n.d.	[131]	

The table is not necessarily complete. Species specificity is shown as published and/or as tested by the authors, but may be limited to the stated species. Recommendations are based on self-assessed observations of the authors. No recommendation does not necessarily mean that the respective siRNA is not recommended, as the authors did not test all siRNAs

*n.s.* not specified, *n.q.* not quantified, *ctr.* control

### NOX knock-out mouse models

**Fig. 2:**
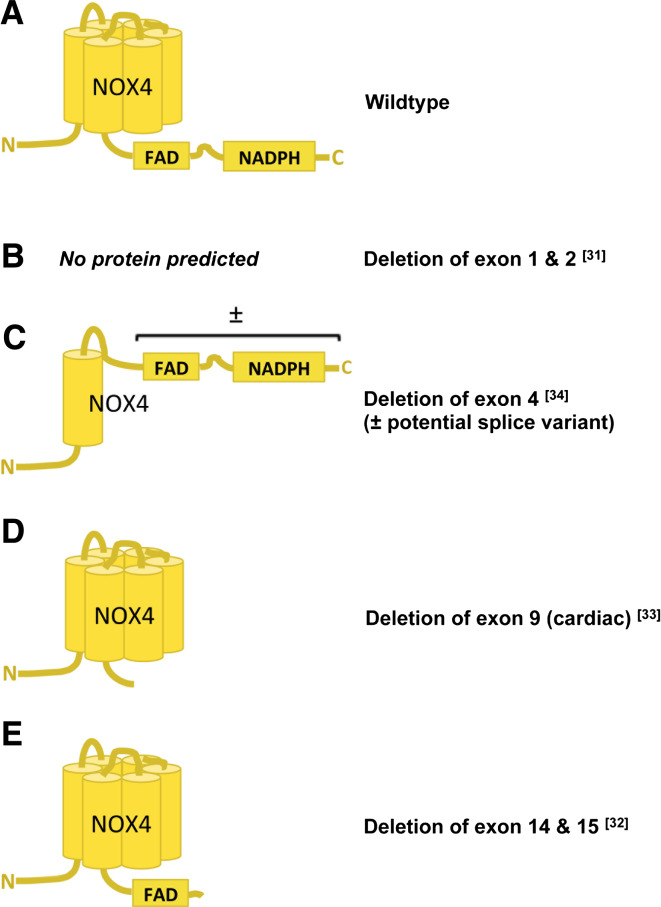
Published NOX4 knock-out (KO) mouse models. **a** Wild-type NOX4 has six transmembrane helices and cytosolic binding domains for FAD and NADPH at the C-terminus. **b** Deletion of exons 1 and 2 should delete the complete NOX4 protein [32]. **c** Deletion of exon 4 only leaves the first transmembrane domain of NOX4. However, hypothetically, this may also result in the formation of a splice variant that contains both FAD and NADPH binding domains and thus has remaining ROS-forming activity [43]. **d** Another knock-out was generated by conditionally deleting exon 9 of NOX4 in cardiomyocytes, thereby deleting the FAD binding domain, likely leaving a non-functional enzyme [34]. **e** The fourth published NOX4 KO mouse was generated by deleting exons 14 and 15 that refer to the NADPH binding domain. This likely results in the expression of a non-functional enzyme [33]

NOX2 knock-out (KO) mice in which exons 2 and 3 are deleted are commercially available [38], and no other NOX2 KO model has been published. Two identical NOX1 KO mice carrying a deletion of exons 3–6 have been published showing a mild hypotensive phenotype and attenuated angiotensin II-induced hypertension [39, 40]. Unfortunately, no western blot data using tissues of these mice to confirm the absence or size of a possibly residual NOX1 protein have been published. An N-terminally truncated or alternatively spliced NOX1 protein may still be expressed [41]. However, it is unlikely that NOX1 splice variants lacking the binding sites for regulatory subunits have any ROS-producing activity. With respect to NOX4, there is more variety, and four NOX4 KO mouse models have been published to date (Fig. 2). All differ in the genetic strategy that was applied to generate them, i.e. different exons were deleted (exons 1/2, exon 4, exon 9, or exons 14/15) and constitutive, cell-specific or inducible cre/lox systems were used. In future, this may also help to elucidate the role of alternative splicing in mouse NOX4 biology [32–35]. Indeed, the possibility exists that, at least in some tissues, the deletion of an early exon may lead to truncated but active NOX4 variants and thus residual NOX4 activity. Interestingly, an analogue to the human NOX4 splice variant D [42] lacking exons 3–11 of murine NOX4 has been found in kidney and colon. Importantly, this 28-kDa NOX4 isoform (Fig. 2c) was still capable of producing ROS, and the authors could blunt this activity by selective siRNA silencing of this particular isoform [43]. This observation is supported by the findings that the isolated NOX4 dehydrogenase domain is still able to reduce substrates like certain artificial dyes [44]. Although not shown directly for NADPH oxidases, it is known that flavin-binding domains are able to reduce oxygen, thus forming superoxide [45, 46]. Accordingly, the residual NADPH- and flavin-containing protein seems to be sufficient to catalyze ROS formation. Only in mice containing a deletion of either exon 9 (FAD binding site) or 14/15 (NADPH binding site) is it unlikely that any residual NOX4 protein could still produce ROS. It is discussed in the field that potential shortened inactive NOX4 proteins present in exon 9 or exons 14/15 deletions exert dominant negative or positive effects on other NOX isoforms (e.g., NOX1 and NOX2) or NOX binding proteins. For example, in the absence of NOX4, more free p22^phox^ may be available to interact with NOX1/2. Such mechanisms could affect both the expression and activity of other NOX isoforms. However, protein levels of other NOX isoforms have not been reported to be altered in NOX4 KO mice [33]. Further, if the activity of other NOX isoforms would be influenced these mice would then be expected to show a mixed phenotype of NOX4 and NOX1 and/or NOX2 KO mice, e.g. reduced blood pressure and angiotensin II-induced pressure response (NOX1; [39, 40]) or impaired oxidative burst activity of circulating neutrophils (NOX2; [38]). The neutrophil phenotype remains to be analyzed. A dominant negative regulation of other NOX isoforms in other cell-types of NOX4 KO cannot be completely ruled out unless studied. The lack of an effect on blood pressure by NOX4 deletion in mice [33] argues against such a hypothetical mixed NOX1/4 phenotype.

### Transgenic NOX4 overexpressing mouse models

Parallel to the NOX4 KO mice, three different transgenic NOX4 (tgNOX4) overexpressing mice have been published, two of a cardiomyocyte-specific manner [32, 36] and the most recent in an endothelial-specific manner [37]. Surprisingly, the endothelial tgNOX4 mouse had a lower systemic blood pressure compared to littermate wild-type mice, which does not match the vascular phenotype of any of the NOX4 KO mice, which are all reported to have unchanged blood pressures [32–34]. Similar to the discussion above on bystander effects on other NOX isoforms in NOX4 KO mice, NOX4 overexpression may also affect both expression and activity of NOX1/2. For example, less p22^phox^ may be available to interact with NOX1/2. However, NOX1 was below detection limits in aortae from both wild-type and tgNOX4 animals, and NOX2 levels were unchanged [37]. Thus, dominant negative effects of a transgenic expression of NOX4 on other NOX isoforms cannot be excluded, but based on all available data are unlikely. The discrepancy in blood pressure might be due to non-physiologically high levels or different subcellular localization of the overexpressed NOX4 compared to endogenous NOX4, a general problem of transgenic overexpression models. A similar subcellular localization of tgNOX4 and endogenous NOX4 was shown in cardiomyocytes [32], but no immunofluorescence data in the endothelium have been published up to date.

### siRNA mediated knock-down of NOX4

There are an increasing number of reports using siRNAs approaches directed against NOX4 (Table 1). Unfortunately, only a few of those siRNAs have been properly validated regarding their overall and NOX isoform specificity. The necessity for confirming specificity was impressively underlined in a recent study [47], which showed that out of nine tested NOX4-directed siRNAs only six down-regulated murine NOX4 mRNA levels. Moreover, five of those six also down-regulated NOX1 mRNA levels. Another problem with investigating the role of NOX4 using siRNAs is the lack of specific antibodies against NOX4. Many if not all publications thus rely primarily on the down-regulation of NOX4 mRNA (see Table 1). These reports may need to be re-evaluated, as it was also recently shown that NOX4 is highly regulated at the post-transcriptional level, and therefore mRNA levels may not necessarily reflect protein levels and ROS formation [48, 49].

### Antibodies against NOX

The lack of specific, freely available and validated antibodies against NOX1 and NOX4 represents one of the biggest roadblocks in the field. As described above, the validation of both siRNA-mediated down-regulation and genetic NOX1 and NOX4 KO models depends on the quality of the antibodies used for the characterization. Furthermore, as long as the tissue distribution of NOX1 and NOX4 remains unclear, it is very difficult to predict or estimate specific versus off-target effects of potential therapeutic interventions. Several groups and companies have attempted to generate polyclonal antibodies directed against different NOX1 and NOX4 peptides or recombinant proteins (Table 2). As these are polyclonal rabbit antibodies, the access and the amount were always limited. Also, several different protein sizes have been detected for NOX4 by different antibodies in the same tissues. This may be due to unspecificity of some antibodies, but also caused by the high sensitivity of the NOX4 protein to lysis conditions that may result in degradation and dephosphorylation [50]. So far, the polyclonal NOX4 antibodies by the Lambeth and Shah groups are the most frequently used. Of those antibodies which we have tested for isoform specificity, we recommend to use the NOX4 antibody from the Shah laboratory [51] and our NOX1 antibody [52]. In 2010, the successful generation of the first monoclonal mouse antibodies against human NOX4 was reported [50]; they were used to analyze the tissue distribution, subcellular localization, and structural features of NOX4 [17, 50]. Two of these antibodies (6B11 and 5F9) moderately block constitutive NOX4 activity in cell-free activity assays [50]. Another monoclonal antibody derived from rabbit is already commercially available, but no data have been published using this antibody in tissues and cells other than monocytes and macrophages [53]. These new antibodies may be promising and freely available tools for the validation of NOX1 and NOX4 as a therapeutic target. For NOX2, the commercially available antibody from Upstate Technologies (now Millipore, USA) is reliable in our hands.

**Table 2:** Antibodies: a selection of published antibodies raised against NOX proteins and their main characteristics (if known)

NOX isoform	Species	Antigen	Type	Size of detected protein in WB (kDa)	Ref./source	Comment
NOX1	Human	aa 480–493	pAb rabbit	n.s.	[132]	
NOX1	Human	aa 544–556	pAb rabbit	63	[133, 134]	
NOX1	Human, rat, mouse	aa 545–561	pAb rabbit	134	[33, 52, 87, 135–138]	Recommended
NOX1	Human, rat, mouse	Various	pAbs		Commercial	Not recommended
NOX1	Rat	aa 543–558	pAb rabbit	75	[130]	
NOX2	Human, rat, mouse	aa 548–560	pAb rabbit	53, 91	Upstate Technologies, BD Biosciences	Ab from upstate recommended for WB, Ab from BD for IF
NOX4	Human	aa 84–101	pAb rabbit	65	[5, 52, 138]	
NOX4	Human	aa 88–102	pAb rabbit	~70	[110, 139–142]	
NOX4	Human	aa 139–154 and 564–578	pAb rabbit	62	[95]	
NOX4	Human	aa 140–153	pAb rabbit	~70	[143]	
NOX4	Human	aa 222–241	mAb	~58 and 65	[17, 50]	
NOX4	Human	aa 251–266	pAb rabbit	~65 and 90	[78, 144]	
NOX4	Human	aa 256–273	pAb rabbit	65	[145, 146]	
NOX4	Human	aa 320–428 (recombinant peptide)	pAb rabbit	65, 80	[20, 93, 103, 105, 108, 115, 126, 136, 147–170]	
NOX4	Human	aa 389–416	mAb	~58 and 65	[50]	
NOX4	Human	aa 392–398	mAb	~58 and 65	[50]	
NOX4	Human	aa 406–578	pAb rabbit	n.s.	[97]	
NOX4	Human	aa 499–511	pAb rabbit	66 and 72	[97, 171]	
NOX4	Human	aa 500–550	mAb rabbit	66	[53]	
NOX4	Human	aa 553–573	pAb rabbit	70	[172]	
NOX4	Human	aa 556–568	pAb rabbit	65	[17, 32, 33, 47, 51, 87, 125, 173]	Recommended
NOX4	Human	aa 556–569	pAb rabbit	64	[42, 92]	
NOX4	Human	aa 558–578	pAb rabbit	n.s.	[105]	
NOX4	Human	aa 559–578	pAb rabbit	66 + 2 bands >94	[97, 98, 101, 174, 175]	
NOX4	Human	aa 564–578	pAb rabbit	n.s.	[176, 177]	
NOX4	Human	n.s.	pAb rabbit	~62	[178, 179]	
NOX4	Mouse	aa 88–103	pAb rabbit	55 and 60	[180, 181]	
NOX4	Mouse	aa 299–515	pAb rabbit	70–75	[131, 182–188]	
NOX4	Mouse	aa 307–578	mAb mouse	~65	[36]	
NOX4	Mouse	aa 553–572	pAb rabbit	n.s.	[189]	
NOX4	Rat	aa 81–95 and 566–578	pAb rabbit	62	[190]	

The table is not necessarily complete. Recommendations are based on self-assessed observations of the authors. No comment does not necessarily mean that the respective antibody is not recommended by the authors, as they have not tested all of them

*WB* western blot, *IF* immunofluorescence, *n.s.* not specified, *aa* amino acid, *pAb* polyclonal antibody, *mAb* monoclonal antibody

### Pharmacological NOX inhibitors

An important tool for the validation of potential therapeutic targets and proof of principle studies is the pharmacological inhibition by small chemical compounds. Several compounds have been used for many years, including apocynin, diphenylene iodonium (DPI), and 4-(2-aminoethyl)-benzensulfonylfluorid (AEBSF). However, it has become apparent that these inhibitors are not specific for NOX [1]. Apocynin cannot be used as selective NADPH oxidase inhibitor due to its direct antioxidant and several off-target effects [54–57]. DPI is a general flavoprotein inhibitor, also inhibiting, for example, xanthine oxidase and eNOS [54, 58, 59], as well as cholinesterases and a calcium pump [60]. AEBSF is primarily a serine protease inhibitor [61]. An ideal NOX-inhibitor would have to fulfil several criteria: it should be active in cell-free conditions, have no intrinsic antioxidant activity, not inhibit other sources of ROS, and ideally be NOX isoform selective. To be applied as a tool for target validation, it should be effective in cells and tissues. For the development into a therapeutic drug, ADME must permit in vivo application and toxicity at an acceptable risk-to-benefit ratio. Recently, several NADPH oxidase-specific and even isoform-specific NOX inhibitors [62–66] have been published; we focus here on the first NADPH oxidase, but not isoform selective inhibitor, VAS2870 and its analogue VAS3947. For a detailed overview of the other interesting compounds, including the highly promising GKT136901, we refer to other publications [1, 64, 67, 68].

## The NADPH oxidase inhibitors VAS2870 and VAS3947

The first published inhibitors that resulted from a systematic screening effort for selective NADPH oxidase inhibitors were the triazolo pyrimidines, represented by the commercially available VAS2870 and its derivatives, such as VAS3947 [69]. VAS3947 shows an improved solubility but does not differ in its inhibition profile (unpublished data). In contrast to formerly used NADPH oxidase inhibitors, the VAS compounds do not show intrinsic anti-oxidant activity nor do they inhibit other flavoproteins such as eNOS and xanthine oxidase [59].

### Validation of the VAS compounds

NADPH oxidase inhibition by VAS2870 and VAS3947 was observed in different cell-free assays including whole cell homogenates of A7r5 (mainly expressing NOX4, VAS3947 IC_50_ of 13 μM) and CaCo-2 (mainly expressing NOX1, VAS3947 IC_50_ of 12 μM) cell lines [59]. The ability to inhibit NOX2 can be concluded from experiments using either intact HL-60 cells (VAS2870 IC_50_ of 1–2 μM) or isolated membranes of human neutrophils containing NADPH oxidase complexes formed from recombinant cytosolic subunits and NOX2 in the presence of SDS (VAS2870 IC_50_ of 10.6 μM) [70, 71]. Furthermore, NADPH oxidase inhibition by VAS inhibitors could be detected in various native, i.e. non-overexpressing, cells expressing different NOX isoforms, including PMA-stimulated human granulocytes (expressing NOX2) [72] and DMSO-differentiated HL60 cells (mainly expressing NOX2) [59], several liver carcinoma cell lines [73], oxLDL-treated human umbilical vein endothelial cells (HUVEC) [74], and PDGF-stimulated primary murine vascular smooth muscle cells [70]. In tissue samples, VAS2870 inhibits ROS release from aortas of aged spontaneous hypertensive rats (SHR) [59]. Also in endothelium-denuded rat tail arteries [75] and in hypoxic mouse brain slices [33], a significant decrease in ROS production was observed after VAS2870 treatment. In a mouse brain ischemia reperfusion model, NADPH oxidase activity was inhibited by in vivo treatment with VAS2870 [33], and in a zebrafish model of wound healing, DUOX was inhibited by VAS2870 [76]. In summary, VAS2870 is a well-validated NADPH oxidase inhibitor, as it shows no intrinsic anti-oxidant activity, does not inhibit other flavoproteins, inhibits NADPH oxidase-mediated ROS production in cell free systems, cells, tissues and in vivo, but it is not NOX isoform-specific. Very recently thioalkylation of cysteine residues of the ryanodine receptor Ca^2+^ channel (RyR1) was discovered as a potential off-target effect of VAS2870 in sarcoplasmic reticulum vesicles isolated in glutathione (GSH) free buffer [193]. The authors also show binding of VAS2870 to low concentrations of GSH in vitro (10 μM). It will be interesting to know to which extent thioalkylation contributes in vivo to the mechanism of action of VAS2870 in the presence of physiological (mM) concentrations of GSH. However, for further development of the compound into a drug more extended off-target effects, ADME and safety data are required, including acute and chronic toxicity determination. So far, it has only been shown that VAS2870 does not inhibit ligand-induced platelet-derived-growth factor receptor (PDGFR)-tyrosine phosphorylation or PDGF-dependent phosphorylation of Erk1/2 or Akt [70].

### Mechanism of action

**Fig. 3:**
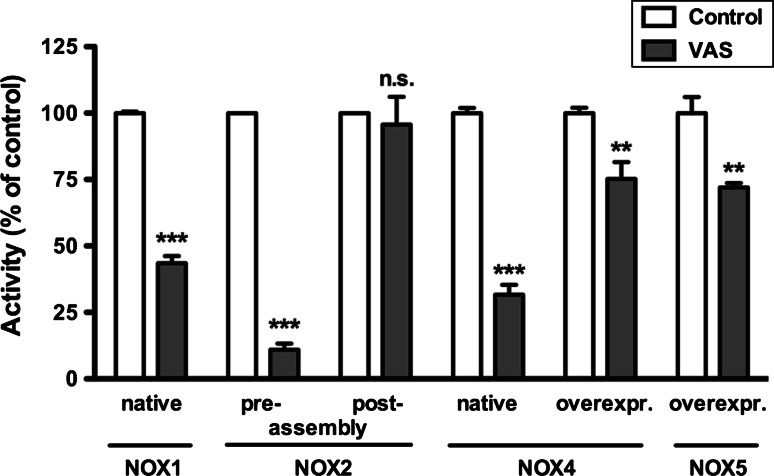
VAS2870 inhibits assembly of NADPH oxidases. *NOX1* whole cell homogenates of CaCo-2 cells (native) were prepared and ROS measured as described in presence or absence of VAS3947 (30 μM) [59]. *Columns* represent means ± SEM of *n* = 3 experiments normalized to untreated controls. *NOX2* membranes of human neutrophils, Rac-2-enriched cytosol fraction as well as recombinant p47^phox^ and p67^phox^ were treated with SDS to induce assembly of these subunits as described [71]. VAS2870 (55 μM) or a solvent control were added before (pre-) or after (post-) assembly of NOX2 with its subunits, and NADPH oxidase activity was measured using the cytochome c reduction assay as described [71]. *Columns* represent means ± SEM of *n* = 3 experiments normalized to untreated controls. *NOX4* whole-cell homogenates of A7r5 cells (native), mainly expressing NOX4 compared to other NOX isoforms, were prepared and ROS measured as described in presence or absence of VAS3947 (30 μM) [59]. *Columns* represent means ± SEM of *n* ≥ 5 experiments normalized to solvent treated controls. Untransfected HEK293 cells (not shown) or HEK293 cells stably transfected with human NOX4 (overexpr.) were treated with VAS2870 or solvent control, and H_2_O_2_ release was measured using Amplex Red. Briefly, Amplex Red (20 μM) and horseradish peroxidase (100 mU/ml) in a phosphate buffer containing VAS2870 (10 μM) or equal volumes of solvent as control were incubated for 10 min at 37 °C in the dark in a 96-well plate. Then, 10^5^ native or human NOX4 overexpressing HEK293 cells were added to the wells, and fluorescence was recorded for 60 min in a Wallac Victor V (Perkin Elmer Life Sciences, Waltham, MA, USA) or Spectramax M2 (Molecular Devices, Sunnyvale, CA, USA) plate reader using 540/590 nm excitation/emission wavelength filters. *Columns* represent means ± SEM of the AUC of time-dependent fluorescence curves in quadruplicates of *n* = 4 experiments normalized to non-VAS treated HEK293-NOX4 cells. *NOX5* L012 (100 μM) was used to measure NOX5 activity in HEK293 cells stably transfected with human NOX5 beta. VAS2870 (10 μM) or a solvent control was added to the cells in a 96-well plate, and basal chemiluminescence was recorded in a Victor V plate reader with 10 readings per well. Then, NOX5 was stimulated with phorbol myristate acetate (PMA, 1 μM) and the calcium ionophore ionomycin (1 μM), or HBSS as control was added (not shown), and chemiluminescence was measured for 20 readings per well for 60 min. *Columns* represent means ± SEM of AUC of time-dependent chemiluminescence in quadruplicates normalized to VAS2870 solvent control. (***p < 0.001, **p < 0.01 are significantly different from control values; *n.s*. *p* > 0.05 not significantly different from control values; 1-way ANOVA calculated with GraphPad Prism5 for each individual experiment)

In a cell-free system (membranes plus cytosol) VAS2870 only inhibited NOX2 activity when added prior to stimulation of the active complex formation between NOX2 and its cytosolic partners [71], whereas it showed no effect on NOX2 activity when added after stimulation of the complex formation with SDS (Fig. 3). This suggests that VAS2870 inhibits NADPH oxidase complex formation and can interfere with the association of NOX and its binding proteins. Surprisingly, the activities of NOX4 and NOX5, that are believed to be independent of cytosolic binding proteins, were also inhibited by VAS2870 when tested in native, mainly NOX4-expressing, A7r5 cells and NOX4 or NOX5 overexpressing HEK-293 cells, respectively (Fig. 3). Also, in vivo data suggest that VAS2870 does inhibit NOX4 in native systems: in a mouse ischemic stroke model, we observed the same protective effect of VAS2870 in the wild-type as by deletion of NOX4. VAS2870 exerted no additional protective effect in NOX4 KO mice [33]. Additionally, in endothelial cells from wild-type mice, pharmacological inhibition with VAS2870 or siRNA against NOX4 inhibited laminar shear stress-induced p38 MAPK activation mediated by hydrogen peroxide [77], and the effect was the same in endothelial cells from NOX4 KO mice (Santiago Lamas, personal communication). Recent data suggest an intramolecular interaction between unique motifs in C-terminus and cytosolic B-loop of NOX4 that forms a tertiary structure and activates H_2_O_2_ production [78, 79]. An intramolecular conformational change may also mediate the calcium-induced activation of NOX5 [31]. Thus, for all NOX isoforms, it is possible that inhibition of inter- or intramolecular conformational changes is a common mechanism of action of VAS2870. Thioalkylation of critical cysteine residues of NOX enzymes by VAS2870 was recently, e.g. the cytosolic B-loop, suggested [193], but the molecular details and binding sites of this remain to be elucidated.

## Applying the tools: validated targets and possible indications

**Fig. 4:**
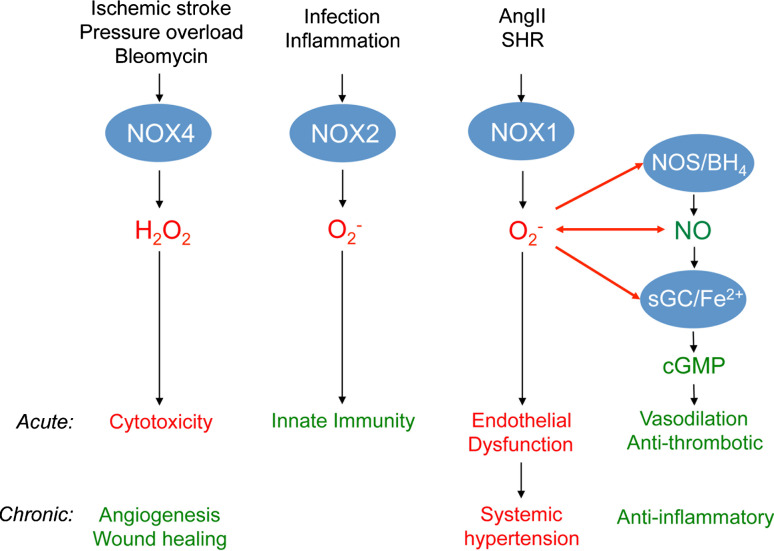
The role of NOX1, NOX2, and NOX4 in disease models. NO, generated by NO-synthases (NOS), activates soluble guanylate cyclase (sGC) by binding to its reduced (Fe^2+^) heme moiety leading to the formation of cGMP from GTP. cGMP mediates protective effects, e.g. vasodilation and anti-inflammation. This signaling pathway is most likely disturbed by NOX1-derived superoxide (O_2_^−^) as shown in Angiotensin II-induced hypertension and spontaneous hypertensive rats (SHR). Superoxide can either directly interact with NO to form peroxynitrite or oxidize the essential NOS cofactor tetrahydrobioapterin (BH_4_) and thus uncouple NOS. Uncoupled NOS forms superoxide itself (not shown). Further, superoxide can oxidize the Fe^2+^ heme of sGC. Thereby, sGC becomes insensitive to NO. These mechanisms most likely account, at least in part, for the acute effects of increased NOX1 activity mediating endothelial dysfunction and the chronic effects that are discussed to cause hypertension. NOX2-derived superoxide is a major signaling molecule in innate immunity mediating host defense. NOX4 is unlikely to directly interfere with the NO/cGMP-signaling pathway as it releases hydrogen peroxide (H_2_O_2_) and not superoxide. However, in high concentrations, H_2_O_2_ causes acute cytotoxicity. This mechanism is suggested to be involved in NOX4-mediated effects after acute ischemic stroke, acute effects of pressure overload in heart, and bleomycin-induced cytotoxicity. The lower chronic activity of NOX4 seems to be involved in angiogenesis and wound healing, and thus rather protective

It is still early days in NOX research, and certainly with respect to translation. Nevertheless, what can already be said about validated roles of NOX and NADPH oxidase in disease? And which of these roles may be translated into therapeutic indications? Different NOX subunits have been suggested to be implicated in cancer, hypertension, lung fibrosis, stroke, heart failure, diabetes, and neurodegenerative diseases [18]. Several principal ways may be differentiated by which an excess of ROS leads to pathology: spatially confined levels of ROS (e.g., in caveolae) that interfere with nitric oxide’s (NO) vasoprotective signaling, and high levels (local or systemic) that act, at least in part, independently of NO and are directly cytotoxic, cause apoptosis (Fig. 4), or disturb redox-sensitive signaling pathways.

### Roles of NOX1, NOX2, and NOX4

NOX2 appears to be relevant in almost every disease model tested. This may be connected to the role of NOX2 in the innate immune response [80], including to fungal infections [81, 82] and adaptive immune response at the level of both T cells and antigen-presenting cells [83, 84]. Thus, in any animal model involving a significant inflammatory response, NOX2 inhibition may lead to an improvement. Whether this can be exploited in light of the essential immune functions of NOX2 is an important question. Importantly, even a small residual NOX2 activity in X-linked chronic granulomatous disease (CGD) is sufficient for a functional innate immune system [85]. However, it is unknown whether a partial pharmacological inhibition of NOX2 will sufficiently suppress NOX2’s non-CGD disease-related activity. In addition, chronic NOX2 inhibition might lead to paradoxical autoimmune responses [86]. Rather, one may want to optimize any NOX inhibition approach by leaving NOX2 unaffected.

With respect to low and spatially confined ROS overproduction, NOX1 is a good candidate to migrate into caveolae and there cause eNOS uncoupling and endothelial dysfunction, which is often associated with increased blood pressure and enhanced platelet aggregation. Moreover, it may be an early step in the development of atherosclerosis. Indeed, basal blood pressure [39], angiotensin-induced hypertension [39, 40], and endothelium-dependent relaxation in spontaneously hypertensive rats [87] depends—to some degree—on NOX1. However, whether such chronic disease indications would ever become realistic for NOX inhibition is highly questionable unless sophisticated patient stratification biomarkers would become available. Phosphorylation of vasodilator-stimulated phosphoprotein (P-VASP) could become such a marker [88].

With respect to higher levels of ROS that act, at least in part, independently of NO and are directly cytotoxic or cause apoptosis, NOX4 is well validated. NOX4 is induced in ischemic stroke, in pressure overload of the heart, and in a bleomycin model of lung epithelial toxicity resulting in lung fibrosis. Whilst the interpretation of the stroke data obtained with NOX4 KO mice is straightforward and was recently confirmed in a tgNOX4 model of brain ischemia showing larger infarct sizes [194], the pressure overload and lung data are less so. In pressure overload, two models have been applied, proximal aortic or thoracic aortic constriction (TAC), and abdominal aortic banding. Both models differ in the time course by which they affect the heart. The latter, less acute model allows for angiogenesis to occur. NOX4 appears to play a double role by contributing to the cardiomyocyte damage (particularly in the acute TAC model [32]) and by facilitating subacute angiogenesis and promoting cardiac function (only observable in the subacute abdominal aortic banding). This may explain why opposing phenotypes were observed in both NOX4 KO mouse models and different disease models. In particular, the TAC model was tested in a cardiomyocyte-specific KO and therefore leaves vascular cell-dependent angiogenesis by definition unaffected. Thus, NOX4 might both acutely damage the cardiomyocyte and subacutely protect the heart by promoting angiogenesis. NOX4 also promotes angiogenesis in vitro as shown using HUVEC [89, 90] and ovarian cancer cells [91]. Whether these effects may be exploited by defining an optimal time window for NOX4 inhibition in situations of acute heart failure or by interfering with tumor angiogenesis remains to be seen, and it needs to be tested by TAC or cancer models in a global KO animal and by applying NOX inhibitors. The situation in the lung is similarly complicated. Here, a role of NOX4 in the pathogenesis of hypoxic pulmonary hypertension was suggested [92], but not confirmed in NOX4 KO mice [33]. Recent data showed that NOX4 deficiency mediated either by NOX4 siRNA [93], NOX4 inhibition, or NOX4 deletion [35] prevents lung fibrosis. However, this observation may be model-dependent as no protection from lung fibrosis was observed in another NOX4 KO mouse using the same model (Weissmann N. and Schmidt H.H.H.W., unpublished observation). Bleomycin induces apoptosis and inflammation in mouse lung epithelial cells [35]. Thus, NOX4 may be relevant in the bleomycin model, but this model may not reflect the wide spectrum of human lung fibrosis (idiopathic, radiation, silicosis, systemic lupus erythematosus, dermatomyositis, sclerodermia, rheumatoid arthritis, pneumoconiosis, acute respiratory distress syndrome, chronic heart failure, drug-induced). Thus, a model-independent role of NOX4 in lung fibrosis needs to be tested in different models of the disease. Even then, the clinical challenge of a life-long therapy with a NOX4 inhibitor would remain. Importantly, all published NOX4 KO models lack a basal phenotype. This is an important observation for the characterization of NOX4 as a therapeutic target, as it indicates that NOX4 inhibition would probably not cause severe complications. The situation may be different when co-morbidities occur and protective roles of NOX4 may well cause side effects. From the current state of knowledge, such potential side effects of sub-chronic and chronic NOX4 inhibition could arise from decreased angiogenesis.

In conclusion, according to the current knowledge, acute ischemic stroke appears to be one of the most promising and safest targets for NOX inhibition. It evades the risk of chronic therapy and the rather double-edged role of NOX4 in heart failure and angiogenesis. Nevertheless, specific, isoform-selective NOX inhibitors and reliable, freely available antibodies will be key in elucidating the full therapeutic potential of NOX in species other than mouse and in different disease models.

## References

[1] Wingler K, Hermans J, Schiffers P, Moens A, Paul M, Schmidt H (2011). NOX 1, 2, 4, 5: counting out oxidative stress. Br J Pharmacol.

[2] Bjelakovic G, Nikolova D, Gluud LL, Simonetti RG, Gluud C (2007). Mortality in randomized trials of antioxidant supplements for primary and secondary prevention: systematic review and meta-analysis. JAMA.

[3] Vivekananthan DP, Penn MS, Sapp SK, Hsu A, Topol EJ (2003). Use of antioxidant vitamins for the prevention of cardiovascular disease: meta-analysis of randomised trials. Lancet.

[4] Kris-Etherton PM, Lichtenstein AH, Howard BV, Steinberg D, Witztum JL (2004). Antioxidant vitamin supplements and cardiovascular disease. Circulation.

[5] Miller AA, Drummond GR, Schmidt HH, Sobey CG (2005). NADPH oxidase activity and function are profoundly greater in cerebral versus systemic arteries. Circ Res.

[6] Shekelle PG, Morton SC, Jungvig LK, Udani J, Spar M, Tu W (2004). Effect of supplemental vitamin E for the prevention and treatment of cardiovascular disease. J Gen Intern Med.

[7] Bjelakovic G, Nikolova D, Simonetti RG, Gluud C (2004). Antioxidant supplements for prevention of gastrointestinal cancers: a systematic review and meta-analysis. Lancet.

[8] Eidelman RS, Hollar D, Hebert PR, Lamas GA, Hennekens CH (2004). Randomized trials of vitamin E in the treatment and prevention of cardiovascular disease. Arch Intern Med.

[9] Dotan Y, Pinchuk I, Lichtenberg D, Leshno M (2009). Decision analysis supports the paradigm that indiscriminate supplementation of vitamin E does more harm than good. Arterioscler Thromb Vasc Biol.

[10] Gallicchio L, Boyd K, Matanoski G, Tao XG, Chen L, Lam TK (2008). Carotenoids and the risk of developing lung cancer: a systematic review. Am J Clin Nutr.

[11] McNally JS, Davis ME, Giddens DP, Saha A, Hwang J, Dikalov S (2003). Role of xanthine oxidoreductase and NAD(P)H oxidase in endothelial superoxide production in response to oscillatory shear stress. Am J Physiol Heart Circ Physiol.

[12] Skulachev VP (1996). Role of uncoupled and non-coupled oxidations in maintenance of safely low levels of oxygen and its one-electron reductants. Q Rev Biophys.

[13] Zhang R, Brennan ML, Shen Z, MacPherson JC, Schmitt D, Molenda CE (2002). Myeloperoxidase functions as a major enzymatic catalyst for initiation of lipid peroxidation at sites of inflammation. J Biol Chem.

[14] Fleming I, Michaelis UR, Bredenkotter D, Fisslthaler B, Dehghani F, Brandes RP (2001). Endothelium-derived hyperpolarizing factor synthase (Cytochrome P450 2C9) is a functionally significant source of reactive oxygen species in coronary arteries. Circ Res.

[15] Vasquez-Vivar J, Kalyanaraman B, Martasek P, Hogg N, Masters BS, Karoui H (1998). Superoxide generation by endothelial nitric oxide synthase: the influence of cofactors. Proc Nat Acad Sci USA.

[16] Brown DI, Griendling KK (2009). Nox proteins in signal transduction. Free Radic Biol Med.

[17] Takac I, Schroder K, Zhang L, Lardy B, Anilkumar N, Lambeth JD (2011). The E-loop is involved in hydrogen peroxide formation by the NADPH oxidase Nox4. J Biol Chem.

[18] Bedard K, Krause KH (2007). The NOX family of ROS-generating NADPH oxidases: physiology and pathophysiology. Physiol Rev.

[19] Opitz N, Drummond GR, Selemidis S, Meurer S, Schmidt HH (2007). The ‘A’s and ‘O’s of NADPH oxidase regulation: a commentary on “Subcellular localization and function of alternatively spliced NOXO1 isoforms”. Free Radic Biol Med.

[20] Lyle AN, Deshpande NN, Taniyama Y, Seidel-Rogol B, Pounkova L, Du P (2009). Poldip2, a novel regulator of NOX4 and cytoskeletal integrity in vascular smooth muscle cells. Circ Res.

[21] Gianni D, Diaz B, Taulet N, Fowler B, Courtneidge SA, Bokoch GM (2009). Novel p47(phox)-related organizers regulate localized NADPH oxidase 1 (NOX1) activity. Sci Signal.

[22] Diaz B, Shani G, Pass I, Anderson D, Quintavalle M, Courtneidge SA (2009). Tks5-dependent, NOX-mediated generation of reactive oxygen species is necessary for invadopodia formation. Sci Signal.

[23] Janiszewski M, Lopes LR, Carmo AO, Pedro MA, Brandes RP, Santos CX (2005). Regulation of NAD(P)H oxidase by associated protein disulfide isomerase in vascular smooth muscle cells. J Biol Chem.

[24] Chen F, Pandey D, Chadli A, Catravas JD, Chen T, Fulton DJ (2011). Hsp90 regulates NADPH oxidase activity and is necessary for superoxide but not hydrogen peroxide production. Antioxid Redox Signal.

[25] Banfi B, Clark RA, Steger K, Krause KH (2003). Two novel proteins activate superoxide generation by the NADPH oxidase NOX1. J Biol Chem.

[26] Geiszt M, Lekstrom K, Witta J, Leto TL (2003). Proteins Homologous to p47phox and p67phox support superoxide production by NAD(P)H Oxidase 1 in colon epithelial cells. J Biol Chem.

[27] Takeya R, Ueno N, Kami K, Taura M, Kohjima M, Izaki T (2003). Novel human homologues of p47phox and p67phox participate in activation of superoxide-producing NADPH oxidases. J Biol Chem.

[28] Sumimoto H (2008). Structure, regulation and evolution of NOX-family NADPH oxidases that produce reactive oxygen species. FEBS J.

[29] Tirone F, Cox JA (2007). NADPH oxidase 5 (NOX5) interacts with and is regulated by calmodulin. FEBS Lett.

[30] Montezano AC, Burger D, Paravicini TM, Chignalia AZ, Yusuf H, Almasri M (2010). Nicotinamide adenine dinucleotide phosphate reduced oxidase 5 (NOX5) regulation by angiotensin II and endothelin-1 is mediated via calcium/calmodulin-dependent, rac-1-independent pathways in human endothelial cells. Circ Res.

[31] Banfi B, Molnar G, Maturana A, Steger K, Hegedus B, Demaurex N (2001). A Ca(2+)-activated NADPH oxidase in testis, spleen, and lymph nodes. J Biol Chem.

[32] Zhang M, Brewer AC, Schroder K, Santos CX, Grieve DJ, Wang M (2010). NADPH oxidase-4 mediates protection against chronic load-induced stress in mouse hearts by enhancing angiogenesis. Proc Natl Acad Sci USA.

[33] Kleinschnitz C, Grund H, Wingler K, Armitage ME, Jones E, Mittal M (2010). Post-stroke inhibition of induced NADPH oxidase type 4 prevents oxidative stress and neurodegeneration. PLoS Biol.

[34] Kuroda J, Ago T, Matsushima S, Zhai P, Schneider MD, Sadoshima J (2010). NADPH oxidase 4 (NOX4) is a major source of oxidative stress in the failing heart. Proc Natl Acad Sci USA.

[35] Carnesecchi S, Deffert C, Donati Y, Basset O, Hinz B, Preynat-Seauve O (2011). A key role for NOX4 in epithelial cell death during development of lung fibrosis. Antioxid Redox Signal.

[36] Ago T, Kuroda J, Pain J, Fu C, Li H, Sadoshima J (2010). Upregulation of NOX4 by hypertrophic stimuli promotes apoptosis and mitochondrial dysfunction in cardiac myocytes. Circ Res.

[37] Ray R, Murdoch CE, Wang M, Santos CX, Zhang M, Alom-Ruiz S (2011). Endothelial NOX4 NADPH oxidase enhances vasodilatation and reduces blood pressure in vivo. Arterioscler Thromb Vasc Biol.

[38] Pollock JD, Williams DA, Gifford MA, Li LL, Du X, Fisherman J (1995). Mouse model of X-linked chronic granulomatous disease, an inherited defect in phagocyte superoxide production. Nat Genet.

[39] Gavazzi G, Banfi B, Deffert C, Fiette L, Schappi M, Herrmann F (2006). Decreased blood pressure in NOX1-deficient mice. FEBS Lett.

[40] Matsuno K, Yamada H, Iwata K, Jin D, Katsuyama M, Matsuki M (2005). NOX1 is involved in angiotensin II-mediated hypertension: a study in NOX1-deficient mice. Circulation.

[41] Arakawa N, Katsuyama M, Matsuno K, Urao N, Tabuchi Y, Okigaki M (2006). Novel transcripts of NOX1 are regulated by alternative promoters and expressed under phenotypic modulation of vascular smooth muscle cells. Biochem J.

[42] Goyal P, Weissmann N, Rose F, Grimminger F, Schafers HJ, Seeger W (2005). Identification of novel NOX4 splice variants with impact on ROS levels in A549 cells. Biochem Biophys Res Commun.

[43] Ben Mkaddem S, Pedruzzi E, Werts C, Coant N, Bens M, Cluzeaud F (2010). Heat shock protein gp96 and NAD(P)H oxidase 4 play key roles in Toll-like receptor 4-activated apoptosis during renal ischemia/reperfusion injury. Cell Death Differ.

[44] Nisimoto Y, Jackson HM, Ogawa H, Kawahara T, Lambeth JD (2010). Constitutive NADPH-dependent electron transferase activity of the NOX4 dehydrogenase domain. Biochemistry.

[45] Kussmaul L, Hirst J (2006). The mechanism of superoxide production by NADH: ubiquinone oxidoreductase (complex I) from bovine heart mitochondria. Proc Nat Acad Sci USA.

[46] Xia Y, Roman LJ, Masters BS, Zweier JL (1998). Inducible nitric-oxide synthase generates superoxide from the reductase domain. J Biol Chem.

[47] Schroder K, Wandzioch K, Helmcke I, Brandes RP (2009). NOX4 acts as a switch between differentiation and proliferation in preadipocytes. Arterioscler Thromb Vasc Biol.

[48] Peshavariya H, Jiang F, Taylor CJ, Selemidis S, Chang CW, Dusting GJ (2009). Translation-linked mRNA destabilization accompanying serum-induced NOX4 expression in human endothelial cells. Antioxid Redox Signal.

[49] Lassegue B, Griendling KK (2010). NADPH oxidases: functions and pathologies in the vasculature. Arterioscler Thromb Vasc Biol.

[50] Zhang L, Nguyen MV, Lardy B, Jesaitis AJ, Grichine A, Rousset F (2011). New insight into the NOX4 subcellular localization in HEK293 cells: First monoclonal antibodies against NOX4. Biochimie.

[51] Anilkumar N, Weber R, Zhang M, Brewer A, Shah AM (2008). NOX4 and NOX2 NADPH oxidases mediate distinct cellular redox signaling responses to agonist stimulation. Arterioscler Thromb Vasc Biol.

[52] Wingler K, Wunsch S, Kreutz R, Rothermund L, Paul M, Schmidt HH (2001). Upregulation of the vascular NAD(P)H-oxidase isoforms NOX1 and NOX4 by the renin-angiotensin system in vitro and in vivo. Free Radic Biol Med.

[53] Lee CF, Qiao M, Schroder K, Zhao Q, Asmis R (2010). NOX4 is a novel inducible source of reactive oxygen species in monocytes and macrophages and mediates oxidized low density lipoprotein-induced macrophage death. Circ Res.

[54] Aldieri E, Riganti C, Polimeni M, Gazzano E, Lussiana C, Campia I (2008). Classical inhibitors of NOX NAD(P)H oxidases are not specific. Curr Drug Metab.

[55] Yu J, Weiwer M, Linhardt RJ, Dordick JS (2008). The role of the methoxyphenol apocynin, a vascular NADPH oxidase inhibitor, as a chemopreventative agent in the potential treatment of cardiovascular diseases. Curr Vasc Pharmacol.

[56] Mora-Pale M, WeÔwer M, Yu J, Linhardt RJ, Dordick JS (2009). Inhibition of human vascular NADPH oxidase by apocynin derived oligophenols. Bioorg Med Chem.

[57] Heumuller S, Wind S, Barbosa-Sicard E, Schmidt HH, Busse R, Schroder K (2008). Apocynin is not an inhibitor of vascular NADPH oxidases but an antioxidant. Hypertension.

[58] O’Donnell BV, Tew DG, Jones OT, England PJ (1993). Studies on the inhibitory mechanism of iodonium compounds with special reference to neutrophil NADPH oxidase. Biochem J.

[59] Wind S, Beuerlein K, Eucker T, Muller H, Scheurer P, Armitage ME (2010). Comparative pharmacology of chemically distinct NADPH oxidase inhibitors. Br J Pharmacol.

[60] Tazzeo T, Worek F, Janssen L (2009). The NADPH oxidase inhibitor diphenyleneiodonium is also a potent inhibitor of cholinesterases and the internal Ca(2+) pump. Br J Pharmacol.

[61] Diatchuk V, Lotan O, Koshkin V, Wikstroem P, Pick E (1997). Inhibition of NADPH oxidase activation by 4-(2-aminoethyl)-benzenesulfonyl fluoride and related compounds. J Biol Chem.

[62] Gianni D, Taulet N, Zhang H, DerMardirossian C, Kister J, Martinez L (2010). A novel and specific NADPH oxidase-1 (NOX1) small-molecule inhibitor blocks the formation of functional invadopodia in human colon cancer cells. ACS Chem Biol.

[63] Brown SJ, Gianni D, Bokoch G, Mercer BA, Hodder P, Rosen HR (2010) Probe report for NOX1 inhibitors. In: Probe Reports from the Molecular Libraries Program, Bethesda 21433358

[64] Laleu B, Gaggini F, Orchard M, Fioraso-Cartier L, Cagnon L, Houngninou-Molango S (2010). First in class, potent, and orally bioavailable NADPH oxidase isoform 4 (NOX4) inhibitors for the treatment of idiopathic pulmonary fibrosis. J Med Chem.

[65] Bhandarkar SS (2009). Fulvene-5 potently inhibits NADPH oxidase 4 and blocks the growth of endothelial tumors in mice. J Clin Invest.

[66] Jaquet V, Marcoux J, Forest E, Leidal KG, McCormick S, Westermaier Y (2011). NOX NADPH oxidase isoforms are inhibited by celastrol with a dual mode of action. Br J Pharmacol.

[67] Sedeek M, Callera G, Montezano A, Gutsol A, Heitz F, Szyndralewiez C (2010). Critical role of NOX4-based NADPH oxidase in glucose-induced oxidative stress in the kidney: implications in type 2 diabetic nephropathy. Am J Physiol Renal Physiol.

[68] Garrido-Urbani S, Jemelin S, Deffert C, Carnesecchi S, Basset O, Szyndralewiez C (2011). Targeting vascular NADPH oxidase 1 blocks tumor angiogenesis through a PPARalpha mediated mechanism. PLoS ONE.

[69] Tegtmeier F, Walter U, Schinzel R, Wingler K, Scheurer P, Schmidt H (2005) Compounds containing a N-heteroaryl moiety linked to fused ring moieties for the inhibition of NAD(P)H oxidases and platelet activation. European Patent 1 598 354 A1.

[70] ten Freyhaus H, Huntgeburth M, Wingler K, Schnitker J, Baumer AT, Vantler M (2006). Novel NOX inhibitor VAS2870 attenuates PDGF-dependent smooth muscle cell chemotaxis, but not proliferation. Cardiovasc Res.

[71] Leusen JH, Fluiter K, Hilarius PM, Roos D, Verhoeven AJ, Bolscher BG (1995). Interactions between the cytosolic components p47phox and p67phox of the human neutrophil NADPH oxidase that are not required for activation in the cell-free system. J Biol Chem.

[72] Schluter T, Steinbach AC, Steffen A, Rettig R, Grisk O (2008). Apocynin-induced vasodilation involves Rho kinase inhibition but not NADPH oxidase inhibition. Cardiovasc Res.

[73] Sancho P, Fabregat I (2011). The NADPH oxidase inhibitor VAS2870 impairs cell growth and enhances TGF-beta-induced apoptosis of liver tumor cells. Biochem Pharmacol.

[74] Stielow C, Catar RA, Muller G, Wingler K, Scheurer P, Schmidt HH (2006). Novel NOX inhibitor of oxLDL-induced reactive oxygen species formation in human endothelial cells. Biochem Biophys Res Commun.

[75] Tsai M-H, Jiang MJ (2010). Reactive oxygen species are involved in regulating alpha1-adrenoceptor-activated vascular smooth muscle contraction. J Biomed Sci.

[76] Niethammer P, Grabher C, Look AT, Mitchison TJ (2009). A tissue-scale gradient of hydrogen peroxide mediates rapid wound detection in zebrafish. Nature.

[77] Breton-Romero R, Orduna CG, Romero N, Sanchez FJ, de Alvaro C, Porras A (2012). Critical role of hydrogen peroxide signaling in the sequential activation of p38 MAPK and eNOS in laminar shear stress. Free Radic Biol Med.

[78] von Lohneysen K, Noack D, Wood MR, Friedman JS, Knaus UG (2010). Structural insights into NOX4 and NOX2: motifs involved in function and cellular localization. Mol Cell Biol.

[79] von Loehneysen K, Noack D, Hayes P, Friedman JS, Knaus UG (2012) Constitutive NADPH oxidase 4 activity resides in the composition of the B-loop and the penultimate C-terminus. J Biol Chem 10.1074/jbc.M111.332494 PMC3308764 22277655

[80] Lam GY, Huang J, Brumell JH (2010). The many roles of NOX2 NADPH oxidase-derived ROS in immunity. Semin Immunopathol.

[81] Babior BM (1999). NADPH oxidase: an update. Blood.

[82] Casimir C, Chetty M, Bohler MC, Garcia R, Fischer A, Griscelli C (1992). Identification of the defective NADPH-oxidase component in chronic granulomatous disease: a study of 57 European families. Eur J Clin Invest.

[83] Williams M, Shatynski K, Chen H (2010). The phagocyte NADPH oxidase (NOX2) regulates adaptive immune response at the level of both T cells and APSs. J Immunol.

[84] Jackson SH, Devadas S, Kwon J, Pinto LA, Williams MS (2004). T cells express a phagocyte-type NADPH oxidase that is activated after T cell receptor stimulation. Nat Immunol.

[85] Kuhns DB, Alvord WG, Heller T, Feld JJ, Pike KM, Marciano BE (2010). Residual NADPH oxidase and survival in chronic granulomatous disease. New Engl J Med.

[86] Sareila O, Kelkka T, Pizzolla A, Hultqvist M, Holmdahl R (2011). NOX2 complex-derived ROS as immune regulators. Antioxid Redox Signal.

[87] Wind S, Beuerlein K, Armitage ME, Taye A, Kumar AHS, Janowitz D (2010). Oxidative stress and endothelial dysfunction in aortas of aged spontaneously hypertensive rats by NOX1/2 is reversed by NADPH oxidase inhibition. Hypertension.

[88] Ibarra-Alvarado C, Galle J, Melichar VO, Mameghani A, Schmidt HH (2002). Phosphorylation of blood vessel vasodilator-stimulated phosphoprotein at serine 239 as a functional biochemical marker of endothelial nitric oxide/cyclic GMP signaling. Mol Pharmacol.

[89] Datla SR, Peshavariya H, Dusting GJ, Mahadev K, Goldstein BJ, Jiang F (2007). Important role of NOX4 type NADPH oxidase in angiogenic responses in human microvascular endothelial cells in vitro. Arterioscler Thromb Vasc Biol.

[90] Xu H, Goettsch C, Xia N, Horke S, Morawietz H, Forstermann U (2008). Differential roles of PKCalpha and PKCepsilon in controlling the gene expression of NOX4 in human endothelial cells. Free Radical Biol Med.

[91] Xia C, Meng Q, Liu LZ, Rojanasakul Y, Wang XR, Jiang BH (2007). Reactive oxygen species regulate angiogenesis and tumor growth through vascular endothelial growth factor. Cancer Res.

[92] Mittal M, Roth M, Konig P, Hofmann S, Dony E, Goyal P (2007). Hypoxia-dependent regulation of nonphagocytic NADPH oxidase subunit NOX4 in the pulmonary vasculature. Circ Res.

[93] Hecker L, Vittal R, Jones T, Jagirdar R, Luckhardt TR, Horowitz JC (2009). NADPH oxidase-4 mediates myofibroblast activation and fibrogenic responses to lung injury. Nat Med.

[94] Zhang F, Jin S, Yi F, Xia M, Dewey WL, Li PL (2008). Local production of O_2_- by NAD(P)H oxidase in the sarcoplasmic reticulum of coronary arterial myocytes: cADPR-mediated Ca^2+^ regulation. Cell Signal.

[95] Maranchie JK, Zhan Y (2005). NOX4 is critical for hypoxia-inducible factor 2-alpha transcriptional activity in von Hippel-Lindau-deficient renal cell carcinoma. Cancer Res.

[96] Simon F, Fernandez R (2009). Early lipopolysaccharide-induced reactive oxygen species production evokes necrotic cell death in human umbilical vein endothelial cells. J Hypertens.

[97] Kuroda J, Nakagawa K, Yamasaki T, Nakamura K, Takeya R, Kuribayashi F (2005). The superoxide-producing NAD(P)H oxidase NOX4 in the nucleus of human vascular endothelial cells. Genes Cells.

[98] Shono T, Yokoyama N, Uesaka T, Kuroda J, Takeya R, Yamasaki T (2008). Enhanced expression of NADPH oxidase NOX4 in human gliomas and its roles in cell proliferation and survival. Int J Cancer J Int Du Cancer.

[99] Zhuang J, Jiang T, Lu D, Luo Y, Zheng C, Feng J (2010). NADPH oxidase 4 mediates reactive oxygen species induction of CD146 dimerization in VEGF signal transduction. Free Radic Biol Med.

[100] Wang Z, Wei X, Zhang Y, Ma X, Li B, Zhang S (2009). NADPH oxidase-derived ROS contributes to upregulation of TRPC6 expression in puromycin aminonucleoside-induced podocyte injury. Cell Physiol Biochem Int J Exp Cell Physiol Biochem Pharmacol.

[101] Weyemi U, Caillou B, Talbot M, Ameziane-El-Hassani R, Lacroix L, Lagent-Chevallier O (2010). Intracellular expression of reactive oxygen species-generating NADPH oxidase NOX4 in normal and cancer thyroid tissues. Endocr Relat Cancer.

[102] Jaulmes A, Sansilvestri-Morel P, Rolland-Valognes G, Bernhardt F, Gaertner R, Lockhart BP (2009). NOX4 mediates the expression of plasminogen activator inhibitor-1 via p38 MAPK pathway in cultured human endothelial cells. Thromb Res.

[103] Cutz E, Pan J, Yeger H (2009). The role of NOX2 and “novel oxidases” in airway chemoreceptor O(2) sensing. Adv Exp Med Biol.

[104] Lee S, Gharavi NM, Honda H, Chang I, Kim B, Jen N (2009). A role for NADPH oxidase 4 in the activation of vascular endothelial cells by oxidized phospholipids. Free Radic Biol Med.

[105] Yamaura M, Mitsushita J, Furuta S, Kiniwa Y, Ashida A, Goto Y (2009). NADPH oxidase 4 contributes to transformation phenotype of melanoma cells by regulating G2-M cell cycle progression. Cancer Res.

[106] Li B, Bedard K, Sorce S, Hinz B, Dubois-Dauphin M, Krause KH (2009). NOX4 expression in human microglia leads to constitutive generation of reactive oxygen species and to constitutive IL-6 expression. J Innate Immun.

[107] Sancho P, Bertran E, Caja L, Carmona-Cuenca I, Murillo MM, Fabregat I (2009). The inhibition of the epidermal growth factor (EGF) pathway enhances TGF-beta-induced apoptosis in rat hepatoma cells through inducing oxidative stress coincident with a change in the expression pattern of the NADPH oxidases (NOX) isoforms. Biochim Biophys Acta.

[108] Pendyala S, Gorshkova IA, Usatyuk PV, He D, Pennathur A, Lambeth JD (2009). Role of NOX4 and NOX2 in hyperoxia-induced reactive oxygen species generation and migration of human lung endothelial cells. Antioxid Redox Signal.

[109] Li S, Tabar SS, Malec V, Eul BG, Klepetko W, Weissmann N (2008). NOX4 regulates ROS levels under normoxic and hypoxic conditions, triggers proliferation, and inhibits apoptosis in pulmonary artery adventitial fibroblasts. Antioxid Redox Signal.

[110] Pedruzzi E, Guichard C, Ollivier V, Driss F, Fay M, Prunet C (2004). NAD(P)H oxidase NOX-4 mediates 7-ketocholesterol-induced endoplasmic reticulum stress and apoptosis in human aortic smooth muscle cells. Mol Cell Biol.

[111] Palozza P, Serini S, Verdecchia S, Ameruso M, Trombino S, Picci N (2007). Redox regulation of 7-ketocholesterol-induced apoptosis by beta-carotene in human macrophages. Free Radic Biol Med.

[112] Lee YM, Kim BJ, Chun YS, So I, Choi H, Kim MS (2006). NOX4 as an oxygen sensor to regulate TASK-1 activity. Cell Signal.

[113] Park HS, Chun JN, Jung HY, Choi C, Bae YS (2006). Role of NADPH oxidase 4 in lipopolysaccharide-induced proinflammatory responses by human aortic endothelial cells. Cardiovasc Res.

[114] Mochizuki T, Furuta S, Mitsushita J, Shang WH, Ito M, Yokoo Y (2006). Inhibition of NADPH oxidase 4 activates apoptosis via the AKT/apoptosis signal-regulating kinase 1 pathway in pancreatic cancer PANC-1 cells. Oncogene.

[115] Clempus RE, Sorescu D, Dikalova AE, Pounkova L, Jo P, Sorescu GP (2007). NOX4 is required for maintenance of the differentiated vascular smooth muscle cell phenotype. Arterioscler Thromb Vasc Biol.

[116] Martin-Garrido A, Brown DI, Lyle AN, Dikalova A, Seidel-Rogol B, Lassegue B (2011). NADPH oxidase 4 mediates TGF-beta-induced smooth muscle alpha-actin via p38MAPK and serum response factor. Free Radic Biol Med.

[117] Tong X, Schroder K (2009). NADPH oxidases are responsible for the failure of nitric oxide to inhibit migration of smooth muscle cells exposed to high glucose. Free Radic Biol Med.

[118] Ha JS, Lim HM, Park SS (2010). Extracellular hydrogen peroxide contributes to oxidative glutamate toxicity. Brain Res.

[119] Ha JS, Lee JE, Lee JR, Lee CS, Maeng JS, Bae YS (2010). NOX4-dependent H_2_O_2_ production contributes to chronic glutamate toxicity in primary cortical neurons. Exp Cell Res.

[120] Sedeek M, Callera GE, Montezano A, Gutsol A, Heitz F, Szyndralewiez C (2010). Critical role of NOX4-based NADPH oxidase in glucose-induced oxidative stress in the kidney—implications in type 2 diabetic nephropathy. Am J Physiol Renal Physiol.

[121] Pietrowski E, Bender B, Huppert J, White R, Luhmann HJ, Kuhlmann CR (2011). Pro-inflammatory effects of interleukin-17A on vascular smooth muscle cells involve NAD(P)H- oxidase derived reactive oxygen species. J Vasc Res.

[122] Fu Y, Zhang R, Lu D, Liu H, Chandrashekar K, Juncos LA (2010). NOX2 is the primary source of angiotensin II-induced superoxide in the macula densa. Am J Physiol Regul Integr Comp Physiol.

[123] Groeger G, Mackey AM, Pettigrew CA, Bhatt L, Cotter TG (2009). Stress-induced activation of NOX contributes to cell survival signalling via production of hydrogen peroxide. J Neurochem.

[124] Naughton R, Quiney C, Turner SD, Cotter TG (2009). Bcr-Abl-mediated redox regulation of the PI3 K/AKT pathway. Leukemia Off J Leukemia Soc Am Leukemia Res Fund UK.

[125] Xiao Q, Luo Z, Pepe AE, Margariti A, Zeng L, Xu Q (2009). Embryonic stem cell differentiation into smooth muscle cells is mediated by NOX4-produced H_2_O_2_. Am J Physiol Cell Physiol.

[126] Mahadev K, Motoshima H, Wu X, Ruddy JM, Arnold RS, Cheng G (2004). The NAD(P)H oxidase homolog NOX4 modulates insulin-stimulated generation of H_2_O_2_ and plays an integral role in insulin signal transduction. Mol Cell Biol.

[127] Basuroy S, Bhattacharya S, Leffler CW, Parfenova H (2009). NOX4 NADPH oxidase mediates oxidative stress and apoptosis caused by TNF-alpha in cerebral vascular endothelial cells. Am J Physiol Cell Physiol.

[128] Meng D, Lv DD, Fang J (2008). Insulin-like growth factor-I induces reactive oxygen species production and cell migration through NOX4 and Rac1 in vascular smooth muscle cells. Cardiovasc Res.

[129] Block K, Eid A, Griendling KK, Lee DY, Wittrant Y, Gorin Y (2008). NOX4 NAD(P)H oxidase mediates Src-dependent tyrosine phosphorylation of PDK-1 in response to angiotensin II: role in mesangial cell hypertrophy and fibronectin expression. J Biol Chem.

[130] Pleskova M, Beck KF, Behrens MH, Huwiler A, Fichtlscherer B, Wingerter O (2006). Nitric oxide down-regulates the expression of the catalytic NADPH oxidase subunit NOX1 in rat renal mesangial cells. FASEB J Off Publ Fed Am Soc Exp Biol.

[131] Colston JT, de la Rosa SD, Strader JR, Anderson MA, Freeman GL (2005). H_2_O_2_ activates NOX4 through PLA2-dependent arachidonic acid production in adult cardiac fibroblasts. FEBS Lett.

[132] Kawahara T, Kuwano Y, Teshima-Kondo S, Takeya R, Sumimoto H, Kishi K (2004). Role of nicotinamide adenine dinucleotide phosphate oxidase 1 in oxidative burst response to Toll-like receptor 5 signaling in large intestinal epithelial cells. J Immunol.

[133] Yoshida L, Nishida S, Shimoyama T, Kawahara T, Rokutan K, Tsunawaki S (2002). Expression of a p67(phox) homolog in Caco-2 cells giving O(2)(-)-reconstituting ability to cytochrome b(558) together with recombinant p47(phox). Biochem Biophys Res Commun.

[134] Chamulitrat W, Schmidt R, Tomakidi P, Stremmel W, Chunglok W, Kawahara T (2003). Association of gp91phox homolog NOX1 with anchorage-independent growth and MAP kinase-activation of transformed human keratinocytes. Oncogene.

[135] Dikalova A, Clempus R, Lassegue B, Cheng G, McCoy J, Dikalov S (2005). NOX1 overexpression potentiates angiotensin II-induced hypertension and vascular smooth muscle hypertrophy in transgenic mice. Circulation.

[136] Dikalov SI, Dikalova AE, Bikineyeva AT, Schmidt HH, Harrison DG, Griendling KK (2008). Distinct roles of NOX1 and NOX4 in basal and angiotensin II-stimulated superoxide and hydrogen peroxide production. Free Radic Biol Med.

[137] Ambasta RK, Kumar P, Griendling KK, Schmidt HH, Busse R, Brandes RP (2004). Direct interaction of the novel NOX proteins with p22phox is required for the formation of a functionally active NADPH oxidase. J Biol Chem.

[138] Miller AA, Drummond GR, Mast AE, Schmidt HH, Sobey CG (2007). Effect of gender on NADPH-oxidase activity, expression, and function in the cerebral circulation: role of estrogen. Stroke.

[139] Vallet P, Charnay Y, Steger K, Ogier-Denis E, Kovari E, Herrmann F (2005). Neuronal expression of the NADPH oxidase NOX4, and its regulation in mouse experimental brain ischemia. Neuroscience.

[140] Li J, Stouffs M, Serrander L, Banfi B, Bettiol E, Charnay Y (2006). The NADPH oxidase NOX4 drives cardiac differentiation: Role in regulating cardiac transcription factors and MAP kinase activation. Mol Biol Cell.

[141] Amara N, Bachoual R, Desmard M, Golda S, Guichard C, Lanone S (2007). Diesel exhaust particles induce matrix metalloprotease-1 in human lung epithelial cells via a NADP(H) oxidase/NOX4 redox-dependent mechanism. Am J Physiol Lung Cell Mol Physiol.

[142] Mouche S, Mkaddem SB, Wang W, Katic M, Tseng YH, Carnesecchi S (2007). Reduced expression of the NADPH oxidase NOX4 is a hallmark of adipocyte differentiation. Biochim Biophys Acta.

[143] Wendt MC, Daiber A, Kleschyov AL, Mulsch A, Sydow K, Schulz E (2005). Differential effects of diabetes on the expression of the gp91phox homologues NOX1 and NOX4. Free Radic Biol Med.

[144] von Lohneysen K, Noack D, Jesaitis AJ, Dinauer MC, Knaus UG (2008). Mutational analysis reveals distinct features of the NOX4-p22phox complex. J Biol Chem.

[145] Sturrock A, Huecksteadt TP, Norman K, Sanders K, Murphy TM, Chitano P (2007). NOX4 mediates TGF-beta1-induced retinoblastoma protein phosphorylation, proliferation, and hypertrophy in human airway smooth muscle cells. Am J Physiol Lung Cell Mol Physiol.

[146] Sturrock A, Cahill B, Norman K, Huecksteadt TP, Hill K, Sanders K (2006). Transforming growth factor-beta1 induces NOX4 NAD(P)H oxidase and reactive oxygen species-dependent proliferation in human pulmonary artery smooth muscle cells. Am J Physiol Lung Cell Mol Physiol.

[147] Hilenski LL, Clempus RE, Quinn MT, Lambeth JD, Griendling KK (2004). Distinct subcellular localizations of NOX1 and NOX4 in vascular smooth muscle cells. Arterioscler Thromb Vasc Biol.

[148] Szöcs K, Lassegue B, Sorescu D, Hilenski LL, Valppu L, Couse TL (2002). Upregulation of NOX-based NAD(P)H oxidases in restenosis after carotid injury. Arterioscler Thromb Vasc Biol.

[149] Peterson JR, Burmeister MA, Tian X, Zhou Y, Guruju MR, Stupinski JA (2009). Genetic silencing of NOX2 and NOX4 reveals differential roles of these NADPH oxidase homologues in the vasopressor and dipsogenic effects of brain angiotensin II. Hypertension.

[150] Martyn KD, Frederick LM, von Loehneysen K, Dinauer MC, Knaus UG (2006). Functional analysis of NOX4 reveals unique characteristics compared to other NADPH oxidases. Cell Signal.

[151] Kondo S, Shimizu M, Urushihara M, Tsuchiya K, Yoshizumi M, Tamaki T (2006). Addition of the antioxidant probucol to angiotensin II type I receptor antagonist arrests progressive mesangioproliferative glomerulonephritis in the rat. J Am Soc Nephrol JASN.

[152] Liu RM, Choi J, Wu JH, Gaston Pravia KA, Lewis KM, Brand JD (2010). Oxidative modification of nuclear mitogen-activated protein kinase phosphatase 1 is involved in transforming growth factor beta1-induced expression of plasminogen activator inhibitor 1 in fibroblasts. J Biol Chem.

[153] Helmcke I, Heumüller S, Tikkanen R, Schröder K, Brandes RP (2009). Identification of Structural Elements in NOX1 and NOX4 Controlling Localization and Activity. Antioxid Redox Signal.

[154] Touyz RM, Mercure C, He Y, Javeshghani D, Yao G, Callera GE (2005). Angiotensin II-dependent chronic hypertension and cardiac hypertrophy are unaffected by gp91phox-containing NADPH oxidase. Hypertension.

[155] Lu X, Murphy TC, Nanes MS, Hart CM (2010). PPAR{gamma} regulates hypoxia-induced NOX4 expression in human pulmonary artery smooth muscle cells through NF-{kappa}B. Am J Physiol Lung Cell Mol Physiol.

[156] Kawahara T, Ritsick D, Cheng G, Lambeth JD (2005). Point mutations in the proline-rich region of p22phox are dominant inhibitors of NOX1- and NOX2-dependent reactive oxygen generation. J Biol Chem.

[157] Sorescu D, Weiss D, Lassegue B, Clempus RE, Szocs K, Sorescu GP (2002). Superoxide production and expression of NOX family proteins in human atherosclerosis. Circulation.

[158] Hwang J, Kleinhenz DJ, Lassegue B, Griendling KK, Dikalov S, Hart CM (2005). Peroxisome proliferator-activated receptor-gamma ligands regulate endothelial membrane superoxide production. Am J Physiol Cell Physiol.

[159] Lee MY, Martin AS, Mehta PK, Dikalova AE, Garrido AM, Datla SR (2009). Mechanisms of vascular smooth muscle NADPH oxidase 1 (NOX1) contribution to injury-induced neointimal formation. Arterioscler Thromb Vasc Biol.

[160] Cucoranu I, Clempus R, Dikalova A, Phelan PJ, Ariyan S, Dikalov S (2005). NAD(P)H oxidase 4 mediates transforming growth factor-beta1-induced differentiation of cardiac fibroblasts into myofibroblasts. Circ Res.

[161] Peng YJ, Nanduri J, Yuan G, Wang N, Deneris E, Pendyala S (2009). NADPH oxidase is required for the sensory plasticity of the carotid body by chronic intermittent hypoxia. J Neurosci Off J Soc Neurosci.

[162] Spurney CF, Knoblach S, Pistilli EE, Nagaraju K, Martin GR, Hoffman EP (2008). Dystrophin-deficient cardiomyopathy in mouse: expression of NOX4 and Lox are associated with fibrosis and altered functional parameters in the heart. Neuromuscul Disord.

[163] de Mochel NS, Seronello S, Wang SH, Ito C, Zheng JX, Liang TJ (2010). Hepatocyte NAD(P)H oxidases as an endogenous source of reactive oxygen species during hepatitis C virus infection. Hepatology.

[164] Hsiai TK, Hwang J, Barr ML, Correa A, Hamilton R, Alavi M (2007). Hemodynamics influences vascular peroxynitrite formation: Implication for low-density lipoprotein apo-B-100 nitration. Free Radic Biol Med.

[165] Widder JD, Guzik TJ, Mueller CF, Clempus RE, Schmidt HH, Dikalov SI (2007). Role of the multidrug resistance protein-1 in hypertension and vascular dysfunction caused by angiotensin II. Arterioscler Thromb Vasc Biol.

[166] Van Buul JD, Fernandez-Borja M, Anthony EC, Hordijk PL (2005). Expression and localization of NOX2 and NOX4 in primary human endothelial cells. Antioxid Redox Signal.

[167] Edderkaoui M, Hong P, Vaquero EC, Lee JK, Fischer L, Friess H (2005). Extracellular matrix stimulates reactive oxygen species production and increases pancreatic cancer cell survival through 5-lipoxygenase and NADPH oxidase. Am J Physiol Gastrointest Liver Physiol.

[168] Vaquero EC, Edderkaoui M, Pandol SJ, Gukovsky I, Gukovskaya AS (2004). Reactive oxygen species produced by NAD(P)H oxidase inhibit apoptosis in pancreatic cancer cells. J Biol Chem.

[169] Lee JK, Edderkaoui M, Truong P, Ohno I, Jang KT, Berti A (2007). NADPH oxidase promotes pancreatic cancer cell survival via inhibiting JAK2 dephosphorylation by tyrosine phosphatases. Gastroenterology.

[170] Byrne JA, Grieve DJ, Bendall JK, Li JM, Gove C, Lambeth JD (2003). Contrasting roles of NADPH oxidase isoforms in pressure-overload versus angiotensin II-induced cardiac hypertrophy. Circ Res.

[171] Yoshida LS, Tsunawaki S (2008). Expression of NADPH oxidases and enhanced H(2)O(2)-generating activity in human coronary artery endothelial cells upon induction with tumor necrosis factor-alpha. Int Immunopharmacol.

[172] Wagner B, Ricono JM, Gorin Y, Block K, Arar M, Riley D (2007). Mitogenic signaling via platelet-derived growth factor beta in metanephric mesenchymal cells. J Am Soc Nephrol JASN.

[173] Spencer NY, Yan Z, Boudreau RL, Zhang Y, Luo M, Li Q (2011). Control of hepatic nuclear superoxide production by glucose 6-phosphate dehydrogenase and NADPH oxidase-4. J Biol Chem.

[174] Shiose A, Kuroda J, Tsuruya K, Hirai M, Hirakata H, Naito S (2001). A novel superoxide-producing NAD(P)H oxidase in kidney. J Biol Chem.

[175] Etoh T, Inoguchi T, Kakimoto M, Sonoda N, Kobayashi K, Kuroda J (2003). Increased expression of NAD(P)H oxidase subunits, NOX4 and p22phox, in the kidney of streptozotocin-induced diabetic rats and its reversibity by interventive insulin treatment. Diabetologia.

[176] Diebold I, Flugel D, Becht S, Belaiba RS, Bonello S, Hess J (2010). The hypoxia-inducible factor-2alpha is stabilized by oxidative stress involving NOX4. Antioxid Redox Signal.

[177] Diebold I, Petry A, Hess J, Gorlach A (2010). The NADPH oxidase subunit NOX4 is a new target gene of the hypoxia-inducible factor-1. Mol Biol Cell.

[178] Goettsch C, Goettsch W, Muller G, Seebach J, Schnittler HJ, Morawietz H (2009). NOX4 overexpression activates reactive oxygen species and p38 MAPK in human endothelial cells. Biochem Biophys Res Commun.

[179] Goettsch C, Goettsch W, Arsov A, Hofbauer LC, Bornstein SR, Morawietz H (2009). Long-term cyclic strain downregulates endothelial NOX4. Antioxid Redox Signal.

[180] Wang Z, Armando I, Asico LD, Escano C, Wang X, Lu Q (2007). The elevated blood pressure of human GRK4gamma A142 V transgenic mice is not associated with increased ROS production. Am J Physiol Heart Circ Physiol.

[181] Li H, Han W, Villar VA, Keever LB, Lu Q, Hopfer U (2009). D1-like receptors regulate NADPH oxidase activity and subunit expression in lipid raft microdomains of renal proximal tubule cells. Hypertension.

[182] Block K, Gorin Y, Abboud HE (2009). Subcellular localization of NOX4 and regulation in diabetes. Proc Nat Acad Sci USA.

[183] Mandal CC, Ganapathy S, Gorin Y, Mahadev K, Block K, Abboud HE (2010). Reactive oxygen species derived from NOX4 mediate BMP2 gene transcription and osteoblast differentiation. Biochem J.

[184] Bondi CD, Manickam N, Lee DY, Block K, Gorin Y, Abboud HE (2010). NAD(P)H oxidase mediates TGF-beta1-induced activation of kidney myofibroblasts. J Am Soc Nephrol JASN.

[185] Block K, Gorin Y, New DD, Eid A, Chelmicki T, Reed A (2010). The NADPH oxidase subunit p22phox inhibits the function of the tumor suppressor protein tuberin. Am J Pathol.

[186] Gorin Y, Block K, Hernandez J, Bhandari B, Wagner B, Barnes JL (2005). NOX4 NAD(P)H oxidase mediates hypertrophy and fibronectin expression in the diabetic kidney. J Biol Chem.

[187] Block K, Gorin Y, Hoover P, Williams P, Chelmicki T, Clark RA (2007). NAD(P)H oxidases regulate HIF-2alpha protein expression. J Biol Chem.

[188] Ribaldo PD, Souza DS, Biswas SK, Block K, Lopes de Faria JM, Lopes de Faria JB (2009). Green tea (Camellia sinensis) attenuates nephropathy by downregulating NOX4 NADPH oxidase in diabetic spontaneously hypertensive rats. J Nutr.

[189] Yang S, Madyastha P, Bingel S, Ries W, Key L (2001). A new superoxide-generating oxidase in murine osteoclasts. J Biol Chem.

[190] Djordjevic T, BelAiba RS, Bonello S, Pfeilschifter J, Hess J, Gorlach A (2005). Human urotensin II is a novel activator of NADPH oxidase in human pulmonary artery smooth muscle cells. Arterioscler Thromb Vasc Biol.

[191] Petry A, Djordjevic T, Weitnauer M, Kietzmann T, Hess J, Gorlach A (2006). NOX2 and NOX4 mediate proliferative response in endothelial cells. Antioxid Redox Signal.

[192] Banfi B, Tirone F, Durussel I, Knisz J, Moskwa P, Molnar GZ (2004). Mechanism of Ca^2+^ activation of the NADPH oxidase 5 (NOX5). J Biol Chem.

[193] Sun QA, Hess DT, Wang B, Miyagi M, Stamler JS (2012). Off-target thiol alkylation by the NADPH oxidase inhibitor 3-benzyl-7-(2-benzoxazolyl)thio-1,2,3-triazolo[4,5-d]pyrimidine (VAS2870). Free Rad Biol Med.

[194] Arimura K, Ago T, Kuroda J, Ishitsuka K, Nishimura A, Sugimori H (2012). Role of NADPH oxidase 4 in brain endothelial cells after ischemic stroke. Stroke.

